# Cytotoxic Potential of Petroleum ether, Ethyl Acetate, Chloroform, and Ethanol Extracts of *Lavandula Coronopifolia *Against Human Breast Carcinoma Cell line (MDA-MB-321)

**DOI:** 10.31557/APJCP.2019.20.10.2943

**Published:** 2019

**Authors:** Ebtesam S Al-Sheddi

**Affiliations:** *Department of Pharmacognosy, College of Pharmacy, King Saud University, Riyadh, Saudi Arabia.*

**Keywords:** Lavandula coronopifolia, anticancer activity, MDA-MB-231, cytotoxicity, morphological alteration

## Abstract

**Background:**

Breast cancer is the most common cause of deaths in women. The search for traditionally used medicinal plants which can serve as non-toxic and affordable anticancer drugs is the need of the hour. This study aimed to investigate the anticancer potential of extracts of *L. coronopifolia *against human breast carcinoma cell line (MDA-MB-321).

**Methods:**

The MDA-MB-231 cells were plated in 96 well plates and exposed to 10-1,000 µg/ml of *L. coronopifolia *for 24 h. The cytotoxic response of different extracts was measured by MTT assay, neutral red uptake (NRU) assay and cellular morphological alterations under the microscope.

**Results:**

A concentration-dependent decrease in the cell viability of MDA-MB-231 cells was observed after the exposure of petroleum ether, ethyl acetate, chloroform, and ethanol extracts of L. coronopifolia. The cell viability was found to be 82%, 89% and 98% at 1000, 500 and 250 µg/ml, respectively in petroleum ether, 37%, 75% and 88% at 1,000, 500 and 250 µg/ml, respectively in ethyl acetate extract, 30%, 35% and 64% at 1,000, 500 and 250 µg/ml, respectively in chloroform extract and 44%, 65% and 82% at 1000, 500 and 250 µg/ml, respectively in ethanolic extract of *L. coronopifolia *exposed MDA-MB-231 cells. The results also exhibited morphological alterations in MDA-MB-231 cells exposed to various extracts. The cells treated with 250- 1000 µg/ml lost their original morphology and cell linkage as compared to control cells.

**Conclusion:**

These preliminary results suggest the promising anticancer potential of petroleum ether, ethyl acetate, chloroform, and ethanol extracts of *L. coronopifolia *against MDA-MB-321 cells. Further studies are required to know the mechanism(s) involved in the cell death.

## Introduction

The cancer has been recognized as second foremost cause of death worldwide after cardiovascular diseases (WHO, 2015). Among women, breast cancer is the most common type of cancer and the major cause of death (Esmailpoor et al., 2019). In developing countries, breast cancer is prominent reason of death and in developed countries, breast cancer is second leading cause of death (Rafieian-Kopaie and Nasri, 2015). In spite of new diagnosis techniques such as mammography and needle sampling, no significant change has been observed in the mortality rate since many decades. The increasing tumor heterogeneity, drug resistance and high cost of available therapeutic methodologies are present concern that are connected with effective treatment of breast cancer (Chakraborty and Rahman, 2012). Although, excessive progresses have been made to understand the pathophysiology of disease and the development towards conventional chemotherapy even though there is still need to explore effective drug to treat the breast cancer (Lukong, 2017). Thus the novel, non-toxic, target oriented chemotherapeutic agents at affordable cost are yet to be achieved. 

For centuries now, plants have been used in nutrition, cosmetics and medicine. Owing to the presence of various bioactive metabolites, medicinal plants exhibit various pharmacological activities viz. antifungal, antibacterial, antioxidant, spasmodic, anticancer etc. (Dragland et al., 2003). Traditional medicinal plants have been proven to play an important role in the treatment of various cancer diseases, since ancient (Bournine et al., 2017). Therapeutically significant bioactive compounds, such as Taxol, Vinblastine, Vinflunine, Vinorelbine, Camptothecin, Vincristine and Vindesine have been explored from plants and are established to treat the various cancer diseases (Greenwell and Rahman, 2015). The genus Lavandula consists of 39 species of flowering plants in family Lamiaceae (Atta-ur-Rahman, 2005). The plants belonging to this genus are mainly are annuals, shrubs and herbaceous plants. Lavandula plants are known for their antioxidant, insecticidal, anti-inflammatory, sedative and spasmodic properties (Wichtl, 1994). *L. augustifolia *exhibited anti-inflammatory and analgesic activities (Valiollah et al., 2003). *L. stoechas *displayed hepato- and neuroprotective properties (Slimen et al., 2015). *L. pubescens *is known for possessing a broad spectrum antimicrobial (Gouda, 2017), anti-inflammatory and hepatoprotective activities (Mousa et al., 2018). *L. coronopifolia Poir*, *L. dentata* and *L. pubescens Decne *are the three species found in Saudi Arabia. These plants have been used in traditional system of medicine. The infusion of flowers of *L. dentata* is used for urine retention and removal of stones from kidney and ureter. Similarly *L. pubescens *is known to relieve headache and cold (Rahman, 2004). The literature survey also reveals *L. coronopifolia *possess antioxidant (Abeer, 2011), antibacterial (Talibi, 2011) and hepatoprotective potential (Farshori et al., 2015). However, the anticancer properties of *L. coronopifolia *have not been reported so far. Hence, this study was designed to explore the anti-cancer effect of various solvent extracts of *L. coronopifolia *on human breast carcinoma cell line (MDA-MB-321).

## Materials and Methods

*Chemicals and consumables*

Cell culture medium (DMEM), Fetal bovine serum, and trypsin-EDTA (0.25%) were procured from Gibco, Invitrogen, USA. Cell culture plates and other plastic wares used in present study were purchased from Nunc. MTT, neutral red dye, other specified chemicals and solvents were obtained from Sigma. 

*Plant material and extractions*

The plant used in present investigation was collected from Shaza Mountains in Saudi Arabia. The plant was authenticated by a taxonomist and a voucher (# 15799) was submitted at the herbarium, Department of Pharmacognosy, College of Pharmacy, King Saud University. The aerial parts of *Lavandula coronopifolia *plant were air dried and grounded to a coarse powder. For the extraction, powdered plant material was macerated in petroleum ether, ethyl acetate, chloroform, and ethanol successively. The procedure was repeated several times. At each stage the filtrate was collected and the solvent was then evaporated to obtain the extracts of respective solvents. 

*Cell culture*

MDA-MB-231, a human breast cancer cell line was originally purchased from Leibniz Institute DSMZ-German Collection of Microorganisms and Cell Cultures. The cell line, MDA-MB-231 was grown in DMEM medium with 10% fetal bovine serum, 0.2% NaHCO3 and 1% antibiotic/antimycotic solution in a CO_2_ incubator at 37^o^C in high humid atmosphere. 

*Experimental design*

MDA-MB-231 cell line was exposed to 10 - 1,000 µg/ml of petroleum ether, ethyl acetate, chloroform, and ethanol extracts of *L. coronopifolia *for 24 h. After the treatment, cytotoxicity assessment of *L. coronopifolia *extracts was done using MTT assay, neutral red uptake assay, and morphological assessments on MDA-MB-231 cell line. 

*Cytotoxicity Assessment of L. coronopifolia extracts (MTT assay)*

Cytotoxic effects of *L. coronopifolia *extracts was performed using MTT assay as described by Mossman et al., 1983. In brief, MDA-MB-231 cells (10,000 in numbers) were seeded in 96 well culture plates and allowed to adhere in CO_2_ incubator for overnight. Then, MDA-MB-213 cells were treated with 10 - 1,000 µg/ml of petroleum ether, ethyl acetate, chloroform, and ethanol extracts of *L. coronopifolia *for 24 h. After exposure, 10 μl/well MTT solution (5 mg/ml) was added and incubated further for 4 h. Supernatant was removed from the wells and DMSO (200 µl) was added in each well and mixed. The absorbance of plate was then read at 550 nm wave length.

*Cytotoxicity Assessment of L. coronopifolia extracts (NRU assay)*

Cytotoxic effects of *L. coronopifolia *extracts was also assessed by NRU assay as described by Borenfreund and Puerner, 1984. In brief, MDA-MB-231 cells (10,000 in numbers) were seeded in 96 well culture plates and allowed to adhere in CO_2_ incubator for overnight. Then, cells were exposed to various concentrations (10 - 1,000 µg/ml) of petroleum ether, ethyl acetate, chloroform, and ethanol extracts of *L. coronopifolia *for 24 h. After the treatment, neutral red (50 μg/ml) was added in wells and further incubated for 3 h. Then, solution was rapidly washed with solution (0.5% formaldehyde and 1% calcium chloride) and the dye was extracted in a solution of 1% acetic acid and 50% ethanol. The absorbance of plate was then read at 550 nm wave length.

**Figure 1:**
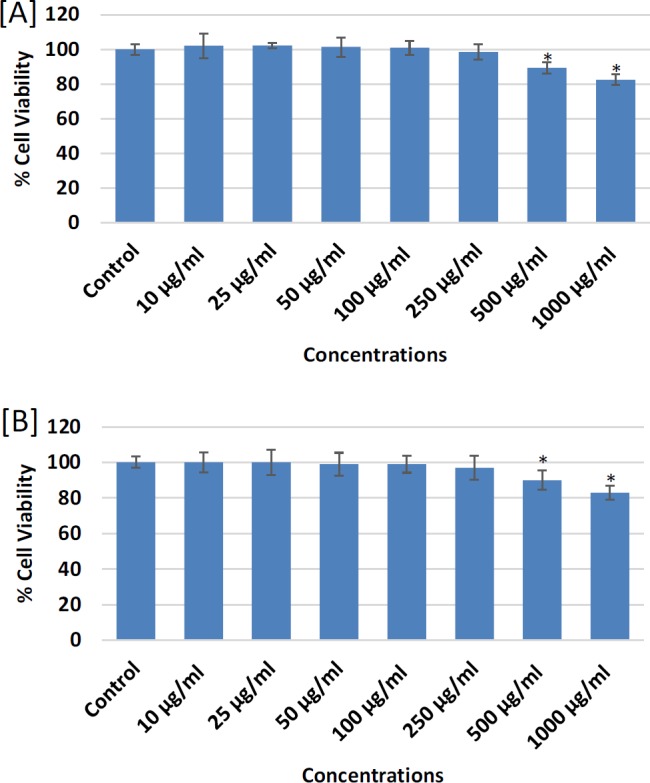
Cytotoxicity Assessments by [A] MTT assay and [B] NRU assay in MDA-MB-231 following the treatment of different concentrations (10-1,000 mg/ml) of petroleum ether extract of *Lavandula coronopifolia* for 24 h. Results are expressed as mean±SD of three independent experiments. (*p<0.05 Vs Control)

**Figure 2:**
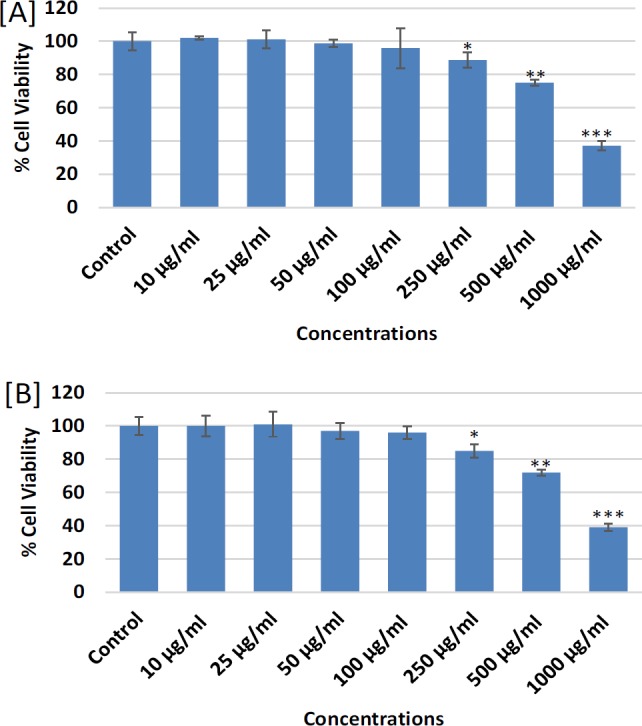
Cytotoxicity Assessments by [A] MTT assay and [B] NRU assay in MDA-MB-231 following the treatment of different concentrations (10-1000 mg/ml) of ethyl acetate extract of *Lavandula coronopifolia* for 24 h. *p<0.05, **p<0.01, ***p<0.001 Vs Control

**Figure 3:**
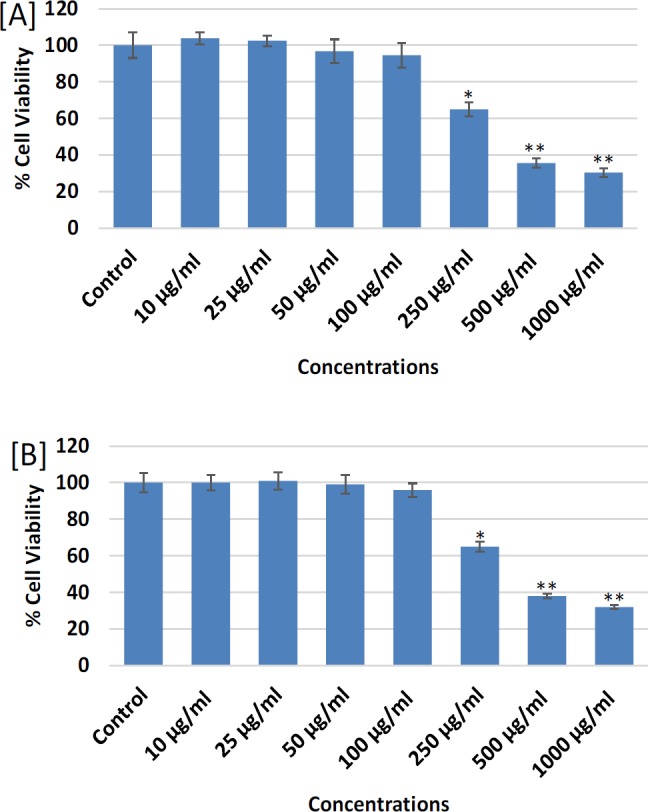
Cytotoxicity assessments by (A) MTT assay and (B) NRU assay in MDA-MB-231 following the treatment of different concentrations (10-1000 mg/ml) of chloroform extract of *Lavandula coronopifolia* for 24 h. *p<0.01, **p<0.001 Vs Control)

**Figure 4:**
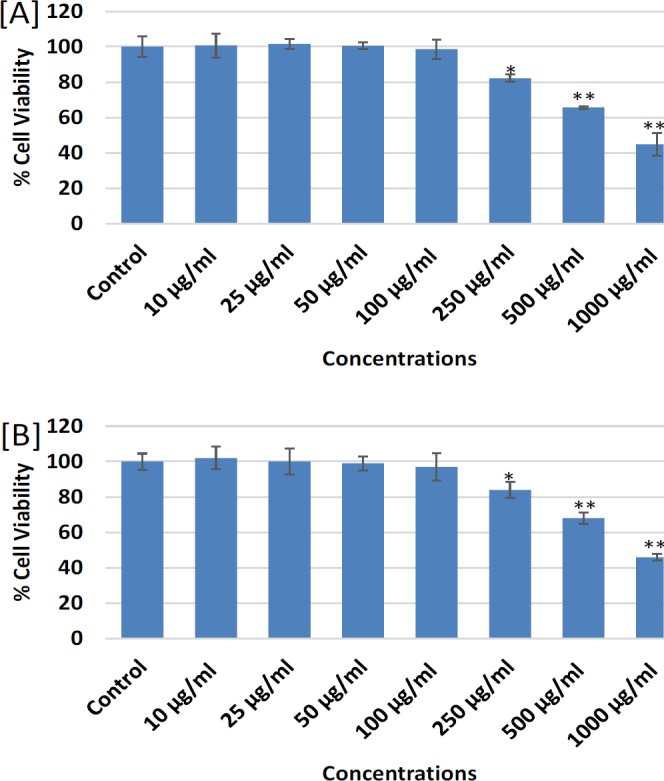
Cytotoxicity assessments by [A] MTT assay and [B] NRU assay in MDA-MB-231 following the treatment of different concentrations (10-1000 mg/ml) of ethanolic extract of *Lavandula coronopifolia* for 24 h. Results are expressed as mean±SD of three independent experiments. *p<0.05, **p<0.01, ***p<0.001 Vs Control

**Figure 5:**
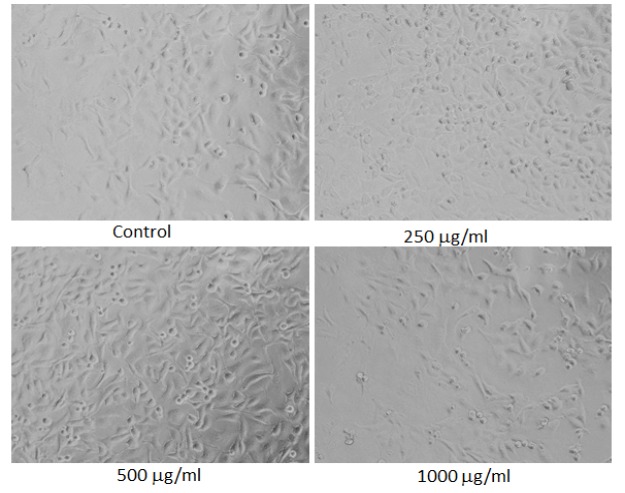
Morphological Changes in MDA-MB-231 Cells Treated to Different Concentrations of Petroleum ether Extract of Lavandula Coronopifolia for 24 h. Images were taken using an inverted phase contrast microscope at 20 X magnification

**Figure 6:**
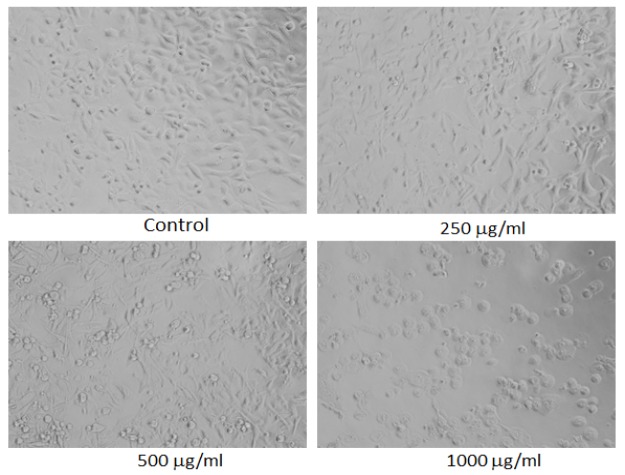
Morphological Changes in MDA-MB-231 Cells Treated to Different Concentrations of Ethyl Acetate Extract of Lavandula Coronopifolia for 24 h. Images were taken using an inverted phase contrast microscope at 20 X magnification

**Figure 7:**
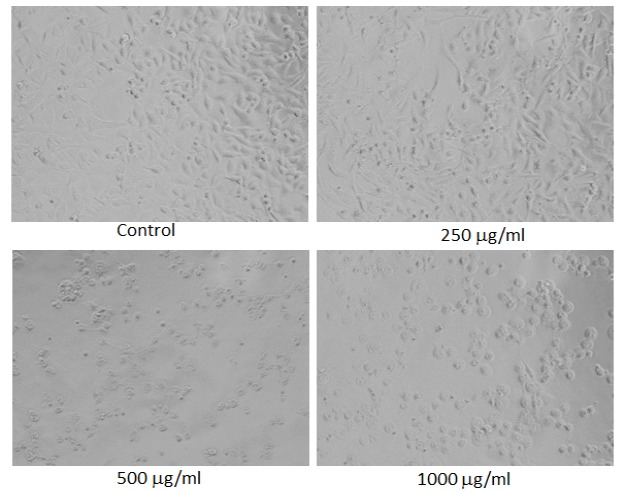
Morphological Changes in MDA-MB-231 Cells Treated to Different Concentrations of Chloroform Extract of Lavandula Coronopifolia for 24 h. Images were taken using an inverted phase contrast microscope at 20 X magnification

**Figure 8:**
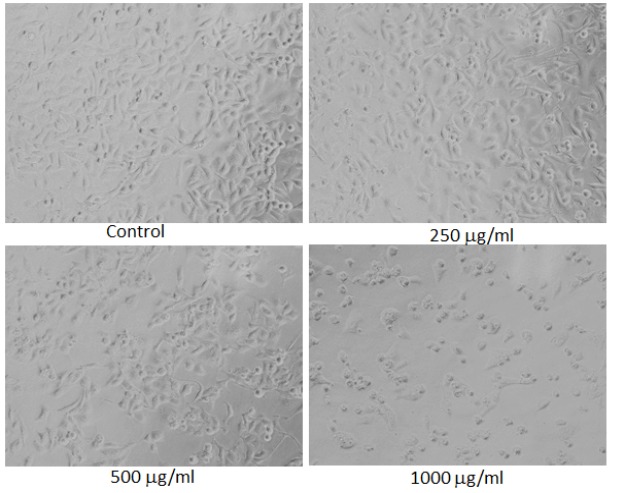
Morphological Changes in MDA-MB-231 Cells Treated to Different Concentrations of Ethanolic Extract of Lavandula Coronopifolia for 24 h. Images were taken using an inverted phase contrast microscope at 20 X magnification

*Morphological analysis*

For the analysis of morphological assessment, the MDA-MB-231 cell line was exposed to 10 - 1,000 µg/ml of petroleum ether, ethyl acetate, chloroform, and ethanol extracts of *Lavandula coronopifolia *for 24 h. After the treatment with Lavandula coronopifolia, cell images of MDA-MB-231 cells were grabbed under the phase contrast inverted microscope at 20× magnification. 

*Statistical analysis*

The p values <0.05 were taken as statistically significant between control and treated groups using One-way ANOVA. The results are expresses as Mean + SD from three independent experiments.

## Results

*Cytotoxicity Assessment of L. coronopifolia extracts (MTT assay)*

The cytotoxic response of petroleum ether, ethyl acetate, chloroform, and ethanol extracts of *L. coronopifolia *against MDA-MB-231 cell line obtained by MTT assay are presented in Figures 1A-4A. MDA-MB-231 cells exposed to various (10-1,000 µg/ml) concentrations of *L. coronopifolia *for 24 h exhibited significant decrease in the cell viability of MDA-MB-231 cell line in a concentration dependent manner. The percent cell viability was found as 82%, 89% and 98% at 1,000, 500 and 250 µg/ml, respectively in petroleum ether extract (Figure 1A), 37%, 75% and 88% at 1,000, 500 and 250 µg/ml, respectively in ethyl acetate extract (Figure 2A), 30%, 35% and 64% at 1,000, 500 and 250 µg/ml, respectively in chloroform extract (Figure 3A) and 44%, 65% and 82% at 1,000, 500 and 250 µg/ml, respectively in ethanolic extract (Figure 4A) of *L. coronopifolia *exposed MDA-MB-231 cells.

*Cytotoxicity Assessment of L. coronopifolia extracts (NRU assay)*

Figures 1B-4B presents the cytotoxic effects of petroleum ether, ethyl acetate, chloroform, and ethanol extracts of *L. coronopifolia *obtained by NRU assay. Like MTT assay, similar kind of cytotoxic effects in MDA-MB-231 cells treated with *L. coronopifolia *extracts were also observed by NRU assay. A concentration dependent cytotoxic effects was observed in MDA-MB-231 cells exposed to 10-1,000 µg/ml for 24 h. The percent cell viability recorded by NRU assay was 83%, 90% and 97% at 1,000, 500 and 250 µg/ml, respectively in petroleum ether extract (Figure 1B), 39%, 72% and 85% at 1,000, 500 and 250 µg/ml, respectively in ethyl acetate extract (Figure 2B), 32%, 38% and 85% at 1,000, 500 and 250 µg/ml, respectively in chloroform extract (Figure 3B) and 46%, 68% and 84% at 1,000, 500 and 250 µg/ml, respectively in ethanolic extract (Figure 4B) of *Lavandula coronopifolia *treated MDA-MB-231 cell line. 

*Lavandula coronopifolia extracts induced morphological alterations in MDA-MB-231 cell line*

*Lavandula coronopifolia *extracts induced morphological alterations in MDA-MB-231 cell line are provided in Figures 5-8. MDA-MB-213 cells exposed to petroleum ether, ethyl acetate, chloroform, and ethanol extracts induced cellular changes in a concentration dependent manner. The alterations in the morphology was observed at 250 µg/ml or above concentrations of *L. coronopifolia *extracts treated for 24 h. At highest concentration, most of the cells lose their original morphology, normal shape and cell linkage capacity as compared to untreated control. After the treatment with the *L. coronopifolia *extracts, MDA-MB-231 cells also became rounded and less in number. 

## Discussion

Breast cancer is one of the vigorous problems and major cause of mortality in female globally. Therefore, the aim of this study was to investigate anticancer potential of *L. coronopifolia *extract against human breast carcinoma (MDA-MB-231) cell line. The MDA-MB-231 cell line in this study was chosen, because it has been proven as a good in vitro model system to investigate the cytotoxicity and anticancer potential of various plant extracts against breast cancer (Alshatwi, 2011; Dilshad et al., 2012, Ghafari et al., 2017; Nasr et al., 2018). Traditionally medicinal plants have been proven to play an important role in the treatment of various cancer diseases (Bournine et al., 2017). Numerous researches have been shown beneficial effects of plant extracts against various cancer cell lines (Pan and Ho, 2008; Farshori et al., 2013, Al-Sheddi et al., 2014). It is well documented that plants and their components could act as cytotoxic/antiproliferative effects and induced apoptosis in various cancer cell lines (Farshori et al., 2014; Al-Oqail et al., 2013). These days, the research on natural/plant phytochemicals are increasing actively because of their potential health benefits and less side effects. In this study, we have chosen *Lavandula coronopifolia *plant because it is reported that *L. coronopifolia *possess antioxidant (Abeer, 2011), antibacterial (Talibi, 2011) and hepatoprotective potential against ethanol induced oxidative stress and cytotoxicity (Farshori et al., 2015). But anticancer properties of *Lavandula coronopifolia *have not been reported against human breast carcinoma cells. The cytotoxic effects of potential of different extracts of *L. coronopifolia *has been assessed using various parameters, such as MTT assay, NRU assay and morphological alterations in MDA-MB-231 cell line treated with different concentrations for 24 h. The MTT and NRU assays showed that petroleum ether, ethyl acetate, chloroform, and ethanol extracts of *L. coronopifolia *decreases the cell viability of MDA-MB-231 cell line in a concentration dependent manner. 

The chloroform extract was found to be more cytotoxic towards MDA-MB231 followed by ethyl acetate extract and ethanolic extract. Petroleum ether extract was found to be less cytotoxic as compared to other extracts. The cytotoxicity of ethyl ether, chloroform and ethanolic extracts were found at 250 µg/ml and above concentrations of Lavandula coronopifolia, whereas, 100 µg/ml and lower concentrations of *Lavandula coronopifolia *did not show significant cytotoxicity to MDA-MB-231 cells. The concentrations range selected in study was chosen from previously reported literature, since exposure of different natural products have been studied by different investigators under in vitro conditions (Maria et al., 1997; Nguta et al., 2012; Solanki and Selvanayagam, 2013). The results of the present study are in accordance to previous report on cytotoxic potential of different plant extracts (Kaneshiro et al., 2005; Kumar et al., 2011). The ethanolic extract of Nigella sativa plant have also been reported to possess cytotoxic effects against P388, HepG2, Molt4 and Lewis lung carcinoma cell lines (Swamy and Tan, 2000). The ethyl acetate extract of Bismillah leaf (Vernonia amygdalina) induced cytotoxicity and morphological alterations have also been reported against human glioblastoma cell line (U-87) (Bin Rohin et al., 2017). In other study, the cytotoxic/antiprolifertaive potential of ethyl acetate extract against HT-29 human colon cancer and MCF-7 human breast cancer cells have also been reported (Lansky and Newman, 2007). The anticancer potential of chloroform extract and sub-fractions of *Nepeta deflersiana *against MCF-7 human breast cancer and A549 human lung carcinoma cell lines have been studied (Al-Oqail et al., 2015). Recently, Usmani et al., (2018) have also reported the antiproliferative and cellular oxidative stress induced by *Cordia dichotoma (Linn.) *extract and its fractions on human cervix epitheloid (HeLa) and human lung (A549) carcinoma cells. The anticancer/cytotoxic effects of *L. coronopifolia *extracts in this investigation suggest that the cytotoxic response of different extracts obtained could be due to the presence of active components, which is showing the anticancer activity of *L. coronopifolia *extracts against human breast cancer cells MDA-MB-231. 

In conclusion, the results from present investigation demonstrated that petroleum ether, ethyl acetate, chloroform, and ethanol extracts of *L. coronopifolia *decreased the cell viability and altered the cell morphology of human breast cancer cell line (MDA-MB-321). The results also showed that chloroform extract was found to be more cytotoxic towards MDA-MB-231 followed by ethyl acetate extract and ethanolic extract. Petroleum ether extract was found to be less cytotoxic as compared to other extracts. Further, more studies are required to know the mechanism(s) of cell death involved in this process. 
